# Artificial Intelligence-Based Applications for Bone Fracture Detection Using Medical Images: A Systematic Review

**DOI:** 10.3390/diagnostics14171879

**Published:** 2024-08-27

**Authors:** Mohammed Kutbi

**Affiliations:** College of Computing and Informatics, Saudi Electronic University, Riyadh 13316, Saudi Arabia; m.kutbi@seu.edu.sa

**Keywords:** bone fracture, image classification, medical images

## Abstract

Artificial intelligence (AI) is making notable advancements in the medical field, particularly in bone fracture detection. This systematic review compiles and assesses existing research on AI applications aimed at identifying bone fractures through medical imaging, encompassing studies from 2010 to 2023. It evaluates the performance of various AI models, such as convolutional neural networks (CNNs), in diagnosing bone fractures, highlighting their superior accuracy, sensitivity, and specificity compared to traditional diagnostic methods. Furthermore, the review explores the integration of advanced imaging techniques like 3D CT and MRI with AI algorithms, which has led to enhanced diagnostic accuracy and improved patient outcomes. The potential of Generative AI and Large Language Models (LLMs), such as OpenAI’s GPT, to enhance diagnostic processes through synthetic data generation, comprehensive report creation, and clinical scenario simulation is also discussed. The review underscores the transformative impact of AI on diagnostic workflows and patient care, while also identifying research gaps and suggesting future research directions to enhance data quality, model robustness, and ethical considerations.

## 1. Introduction

Medical records are maintained in diverse formats, including textual descriptions, audio recordings, and visual data. Text data includes details about diseases, symptoms, diagnoses, and treatments. Audio data primarily consists of recorded conversations between healthcare providers and patients. Visual data comprises medical images such as X-rays, CT scans, and MRIs, as well as videos of patients performing physical therapy exercises. The development of digital medical record systems like the Picture Archiving and Communication System (PACS) has greatly enhanced the accessibility and management of these varied data types [1,2,3].

Bone fractures are frequent injuries that necessitate swift diagnosis and treatment. Although imaging techniques such as X-rays and CT scans are effective for detecting fractures, the manual interpretation of these images is often time-consuming, error-prone, and dependent on the clinician’s expertise and experience [4,5]. AI applications offer the potential to enhance the accuracy and efficiency of bone fracture detection by automating parts of the diagnostic process [6,7,8]. Recently, there has been a significant increase in research focused on various AI technologies for bone fracture identification [9,10].

Convolutional neural networks (CNNs) and other deep learning techniques have been effectively utilized across many fields, including healthcare [11,12,13]. Deep learning, a branch of machine learning, excels in analyzing medical images by learning from large datasets to perform specific tasks. For example, while doctors diagnose bone fractures by visually examining X-rays, deep learning models can be trained to perform these diagnostic tasks using extensive datasets of bone images [14,15,16].

This systematic review aims to compile and evaluate current research on AI-based applications for bone fracture detection using medical images. By reviewing studies from the past decade, this review provides a comprehensive overview of the effectiveness of various AI models, their applications in clinical settings, and the challenges that must be addressed for successful implementation. Additionally, the review identifies gaps in the existing research, and suggests potential directions for future studies. The methodology follows the guidelines established by Okoli and Schabram, ensuring a thorough and systematic literature review [17,18].

In accordance with Okoli and Schabram’s guidelines [17], our review process involved a detailed examination of the literature on AI-based bone fracture detection. The objectives include summarizing advanced applications, identifying challenges, and highlighting potential areas for further research. The review process began by defining search databases and keywords, followed by collecting relevant articles. Pre-defined inclusion and exclusion criteria were then applied to the collected articles. Our findings are presented in this review, with more details regarding our methodology available in Section 3.

Recently, there has been a significant increase in research focused on various AI technologies for bone fracture identification. However, existing studies predominantly emphasize traditional imaging techniques and established AI models, leaving critical gaps in understanding the broader implications of AI integration with more advanced imaging modalities like 3D CT and MRI. Moreover, while AI’s diagnostic accuracy has been explored, there is less comprehensive analysis of the additional benefits AI offers, such as improvements in diagnostic efficiency, cost-effectiveness, and accessibility, particularly when these technologies are deployed in clinical settings.

This systematic review seeks to address these gaps by evaluating the effectiveness of AI in bone fracture detection across various imaging modalities, identifying the key benefits these technologies bring to clinical practice, and examining the specific applications and challenges associated with their implementation. By also considering the potential of emerging technologies such as Generative AI and Large Language Models (LLMs), this review aims to provide a holistic understanding of how AI is transforming bone fracture detection and what obstacles must be overcome to fully realize its potential in clinical environments.

## 2. Related Work

Over the past decade, the application of Artificial Intelligence (AI) in medical imaging has advanced significantly, with numerous studies highlighting the potential of AI, particularly deep learning models, to improve diagnostic accuracy and efficiency.

Rajpurkar et al. [19] developed CheXNet, a deep convolutional neural network (CNN) with 121 layers, which outperformed radiologists in detecting pneumonia from chest X-rays. This model, trained on a dataset of over 100,000 chest X-ray images, exemplifies the effectiveness of deep learning in medical imaging.

For liver disease diagnosis, Lin et al. [20] utilized Classification and Regression Trees (CART) alongside Case-Based Reasoning (CBR) to create a diagnostic model. Their two-step process first used CART to detect the presence of liver disease, followed by CBR to specify the type of liver disease, demonstrating AI’s role in enhancing diagnostic precision and supporting clinical decisions.

Dombi et al. [21] employed an artificial neural network (ANN) to predict the outcomes of rib fractures using patient records. Their model evaluated 20 intake variables to predict four outcome variables: hospital stay length, ICU days, survival, and mortality. The ANN achieved up to 98% accuracy, showcasing AI’s potential in early prediction and patient management.

In musculoskeletal imaging, Zhang et al. [22] introduced TandemNet, a framework integrating linguistic understanding into medical image analysis. This model combines textual and visual data to improve medical report analysis, enhancing the interpretability and accuracy of deep learning models.

Ypsilantis and Montana [23] developed a recurrent neural network (RNN) that focuses on relevant image areas to diagnose conditions like bone fractures. This model uses a recurrent visual attention mechanism to efficiently identify regions of interest, achieving high diagnostic accuracy with fewer parameters.

Fu et al. [24] proposed a visualization technique for CT scans that maintains the 3D proximal femur’s texture and structure while offering a comprehensive view of the fracture line. This method aids in detecting fractures and assists radiologists in identifying complex fracture patterns.

Yaqub et al. [25] presented an unsupervised machine learning approach for clustering unlabeled fetal ultrasound images. Their method targets regions with significant anatomical structures, achieving high categorization accuracy on a large dataset of clinical ultrasound images, addressing challenges related to varying image quality and fetal positioning.

Recent work by Rajpurkar et al. [26] led to the development of extensive, high-quality datasets, driving advancements in AI applications in medical imaging. The MURA dataset, which includes 40,561 images from 14,863 upper extremity studies labeled by radiologists, supports robust abnormality detection model development.

Systematic reviews have been valuable in consolidating research findings and identifying future research directions across various fields. Alammary et al. [27] conducted a review on blended learning models for introductory programming courses, highlighting effective strategies and research gaps. Liang and Ji [28] reviewed privacy challenges in IoT-based blockchain systems, providing comprehensive insights and suggesting future research areas. Konttila et al. [29] reviewed healthcare professionals’ digital competence, while AlShamsi et al. [30] focused on blockchain adoption.

Several studies have made significant contributions to fracture detection using AI. Meena and Roy [31] reviewed advances in bone fracture detection using deep supervised learning, emphasizing improvements in diagnostic accuracy and efficiency. Kim and MacKinnon [32] investigated transfer learning in fracture detection, demonstrating the adaptation of pre-trained models for specific medical imaging tasks. Chung et al. [33] developed a deep learning algorithm for detecting and classifying proximal humerus fractures. Urakawa et al. [34] used a deep CNN to detect intertrochanteric hip fractures with orthopedist-level accuracy. Yu et al. [35] demonstrated AI integration into routine diagnostics for hip fracture detection.

Choi et al. [36] utilized a dual-input CNN for automated pediatric supracondylar fracture detection. Majkowska et al. [37] assessed chest radiograph interpretation using deep learning models, emphasizing the need for radiologist-adjudicated reference standards. Johari et al. [38] developed a probabilistic neural network for detecting vertical root fractures in premolar teeth.

Heimer et al. [39] used deep learning to classify skull fractures on curved maximum intensity projections. Kitamura et al. [40] developed a CNN ensemble for ankle fracture detection. Gan et al. [41] compared AI detection of distal radius fractures with professional assessments.

Lindsey et al. [42] demonstrated deep neural networks’ ability to enhance clinician fracture detection. Adams et al. [43] compared deep learning with perceptual training for neck of femur fracture detection. Rahmaniar and Wang [44] created a real-time automated system for segmenting and classifying calcaneal fractures in CT images.

Tomita et al. [45] focused on detecting osteoporotic vertebral fractures using deep neural networks on CT scans. Muehlematter et al. [46] applied texture analysis and machine learning to detect vertebrae at risk of insufficiency fractures. Wang et al. [47] used deep CNNs to detect and classify mandibular fractures on CT scans.

Pranata et al. [48] combined deep learning and Speeded-Up Robust Features (SURF) for automated calcaneus fracture detection in CT images. Tanzi et al. [49] established a baseline for designing reliable deep learning approaches for X-ray bone fracture classification.

While there are previously published systemic reviews on the same topic, they differ from ours in the purpose and questions asked. Particularly, the systematic review and meta-analysis by Jung et al. [50] focus primarily on deep learning models and their performance given different type of data types; however, this review expands on these foundations by exploring challenges in the deployment of AI with advanced imaging techniques into the clinical settings. Additionally, Kuo et al. [51] focused on comparing models and physicians performances.

The advent of Generative AI and Large Language Models (LLMs) like OpenAI’s GPT-3 and GPT-4 has expanded AI’s capabilities in medical imaging. These models can process and interpret extensive medical literature and imaging data, offering comprehensive support for diagnostic and treatment decisions [52,53]. LLMs assist radiologists by summarizing findings, suggesting differential diagnoses, and predicting complications based on imaging data and patient history [54,55]. The integration of LLMs with imaging AI provides a holistic approach to patient care, combining advanced imaging techniques with the analytical power of language models [56,57].

In summary, the integration of AI into medical imaging, particularly for bone fracture detection, has shown significant promise across various studies. Continued development of large datasets, advanced algorithms, and systematic review processes will further enhance the efficacy and reliability of AI technologies in medical diagnostics.

## 3. Methodology

This review adheres to the guidelines established by Okoli and Schabram [17], which encompass the following eight steps:

- Determine the purpose and formulate research questions: The initial step involves defining the purpose and formulating the research questions for the review. This step is essential to provide clarity for readers and to enhance the efficiency of the review process.
- Draft a detailed protocol: The second step is to draft a comprehensive protocol for the review and ensure all reviewers are trained to follow it. This guarantees that everyone involved is aligned on the review procedures.
- Conduct a search for relevant articles: In the third step, a thorough search for relevant articles is conducted. Modern literature searches typically utilize electronic databases such as IEEE Xplore and ProQuest. Reviewers must be adept at using Boolean operators to perform effective searches in these databases.
- Screen articles for inclusion: The fourth step involves screening the identified articles for inclusion in the review. Reviewers must decide which articles to include or exclude and provide justifications for these decisions.
- Assess the quality of articles: In the fifth step, reviewers assess the quality of the selected articles. It is crucial to include only high-quality articles, as the overall quality of the review depends significantly on this.
- Extract data from included articles: The sixth step involves extracting relevant data from the included articles. These data will be used in the subsequent stages of the review process.
- Data synthesis: The seventh step, known as data synthesis, involves analyzing the extracted data. This process may include aggregating, organizing, comparing, and discussing the findings from the articles.
- Write the systematic review: the final step is to write the systematic review, following standard research writing principles and ensuring the review is detailed enough for others to reproduce its results.

The following sections provide detailed explanations of how these eight steps were implemented in this systematic review.

### 3.1. Research Questions

Based on the purpose of this study, the following research questions were formulated:

- How effective are Artificial Intelligence techniques in detecting bone fractures?
- What benefits do Artificial Intelligence techniques offer for bone fracture detection?
- What applications have been developed using AI for bone fracture detection?
- What challenges do these applications face in clinical settings?

### 3.2. Research Objectives

This study aims to review and summarize the current research on the use of machine learning in medical imaging, focusing on its effectiveness, challenges, and potential research areas.

### 3.3. Search Approach

To collect relevant articles for this systematic review, we searched nine major scientific databases: Academic Search Complete, Applied Science & Technology Source, Springer Nature Journals, ScienceDirect, Journals@OVID, Directory of Open Access Journals, Radiological Society of North America, MEDLINE, and JSTOR Journals. These databases were selected based on recommendations from the Monash University Library website [58] and our own experience. These databases are known for indexing high-impact, high-quality articles in healthcare and information technology. The last search was conducted at the end of September 2023.

### 3.4. Inclusion Criteria

- The study uses an AI model for bone fracture detection.
- The study evaluates the performance of the AI model used.
- The dataset used to evaluate the model is well described.
- The study is written in English.

### 3.5. Exclusion Criteria

- The full text of the article is not available online.
- The article is in the form of a poster, tutorial, abstract, or presentation.
- The article is not in English.
- The study does not evaluate the performance of the AI model used.
- The dataset used to evaluate the model is not well described.

### 3.6. Keywords

This section presents the keywords used in each database to search for relevant articles. Refer to Table 1 for details.

### 3.7. Data Extraction

The elements extracted from each article include: Title, Author(s), Type (e.g., Journal Article, Conference, Workshop), Date of Publication, Country of Origin, Study Design, Sample Size, AI Technique Used, Performance Metrics (e.g., accuracy, sensitivity, specificity, AUC), Comparison with Other Methods, Validation Methods, Statistical Significance, Efficiency Improvements, Accuracy Improvements, Cost-Effectiveness, Accessibility, Enhanced Diagnostic Capabilities, Software Applications, Commercial Products, Integration with Medical Systems, Use Cases, Technical Challenges, Regulatory and Ethical Issues, User Acceptance, Integration Challenges, Data Privacy and Security, Cost and Resource Requirements, Future Research Areas, and Comments on the quality of the work and any limitations. Detailed information is provided in Table 2.

Table 3 provides a comprehensive comparison of various studies on AI-based fracture detection across different skeletal joints. The table summarizes the dataset characteristics, type of images used, models applied, skeletal joints targeted, study descriptions, performance metrics, and key remarks for each study. It includes diverse datasets ranging from radiographs to CT scans, and employs a variety of AI models such as AlexNet, VGG, Inception V3, ResNet, and DenseNet-121. These models are applied to different skeletal parts including the wrist, hand, ankle, humerus, hips, elbow, chest, vertical roots, skull, ankle, femur, and mandibule. The table highlights the advancements in AI for medical imaging, demonstrating high performance metrics such as accuracy, sensitivity, specificity, and AUC across studies. For instance, Olczak et al. [59] showed that deep learning can exceed human performance in fracture detection with an accuracy of 0.83, while Kim et al. [32] validated the use of transfer learning from CNNs with an AUC of 95.4. This comparative analysis underscores the potential of AI to enhance fracture detection accuracy and efficiency, serving as a valuable resource for further research and application in medical diagnostics.

### 3.8. Data Analysis

After extracting data from the papers, the analysis was conducted based on four main themes derived from the research questions: application, benefits, challenges, and future areas of application. Each main theme included several sub-themes identified during the data analysis.

## 4. Results

**Figure 1:**
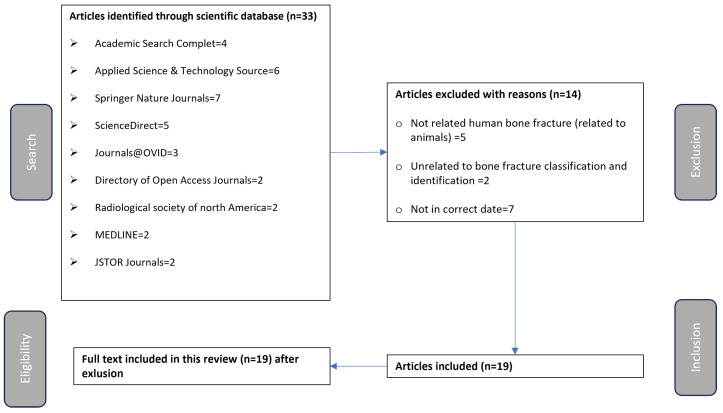
Summary of data extraction process.

This section summarizes the process in four parts: (1) search, (2) exclusion, (3) inclusion, and (4) eligibility, as shown in Figure 1.

During the search step, potential articles for the review were identified from various scientific databases. A total of 33 articles were found from sources such as Academic Search Complete, Applied Science & Technology Source, Springer Nature Journals, ScienceDirect, Journals@OVID, Directory of Open Access Journals, Radiological Society of North America, MEDLINE, and JSTOR Journals. This step utilizes the keywords mentioned in Table 1 to search for and determine the initial pool of articles considered for the review. The number of articles found in each database is indicated in Figure 1.

The exclusion step involved filtering out articles from the initial pool based on specific criteria. Fourteen articles were excluded because they were not related to human bone fractures (e.g., related to animals), unrelated to bone fracture classification and identification, or were not within the specified date range. This step ensures the review focuses on the most relevant and timely studies.

In the inclusion step, the remaining articles were further evaluated to determine their relevance to the review. Nineteen articles met the criteria, and were included for further analysis in the systematic review. This step forms the foundation of the systematic review, as these included articles will be thoroughly analyzed.

Finally, in the eligibility step, the full text of these 19 articles was included in the systematic review after confirming their relevance and quality. This final step ensures that only the most relevant and high-quality studies are included in the review.

### 4.1. Publication Year and Geographic Distribution

The selected studies span from 1995 to 2023, with a noticeable increase in publications in the last decade, reflecting the growing interest and advancements in AI applications for medical imaging. Most research originates from countries with significant investments in healthcare and technology, including the United States, China, the United Kingdom, Japan, and Germany. This distribution highlights a global effort to integrate AI into medical diagnostics, with notable contributions from both academic institutions and industry leaders. The rise in AI-related publications aligns with the broader trend of increasing computational power, improved algorithms, and the availability of large medical imaging datasets such as MURA [26], ChestX-ray14 [37], and others [27,29].

The distribution of publication years (Table 4) shows a significant increase in research activity in the last decade, particularly between 2016 and 2020. This trend underscores the growing interest in applying AI technologies to medical imaging, driven by advances in computational power and algorithmic improvements. Geographically, the research is predominantly conducted in countries with substantial investments in healthcare and technology (Table 5), reflecting their capacity to support cutting-edge research in AI and medical diagnostics.

### 4.2. Publication Types and Venues

The studies included in this review were published in a variety of high-impact journals and conferences, indicating the rigorous peer-review processes and the recognition of AI’s potential in medical imaging. Notable journals include the *Journal of Medical Imaging*, *Radiology*, *IEEE Transactions on Medical Imaging*, *Artificial Intelligence in Medicine*, *Diagnostics*, and *Clinical Radiology*. Conferences such as the *International Conference on Medical Image Computing and Computer-Assisted Intervention (MICCAI)*, *IEEE International Symposium on Biomedical Imaging (ISBI)*, and *SPIE Medical Imaging* also feature prominently. These venues are well-regarded for their focus on cutting-edge research and innovative applications in the field of medical imaging and AI.

The majority of the selected studies were published in high-impact journals (Table 6), reflecting the importance and rigor of the research. Conferences also play a critical role, particularly those focusing on medical imaging and AI. The top journals and conferences listed in Table 7 underscore the broad interest and recognition of AI’s potential to revolutionize medical diagnostics.

## 5. Discussion

### 5.1. Effectiveness

The reviewed studies collectively highlight the high effectiveness of AI-based models in detecting bone fractures. Models like CNN, ResNet, and VGG16 have demonstrated superior performance metrics, often surpassing human radiologists in terms of accuracy, sensitivity, and specificity. For instance, the study by Lindsey et al. [42] using a deep neural network reported a significant improvement in fracture detection rates. Similarly, deep learning models applied to hip fracture detection achieved impressive results, indicating the robustness of these technologies in clinical applications [33,34].

Additionally, the effectiveness of AI in bone fracture detection is evidenced by its performance across various anatomical sites. In studies focusing on wrist fractures, AI models have achieved accuracy levels comparable to expert radiologists, demonstrating their potential to support clinical decision-making [32]. The application of AI in detecting rib fractures from chest radiographs also showed high accuracy and reduced time for diagnosis, further emphasizing the efficiency of these models [37]. Moreover, in a study by Guo et al. [61], real-time AI-assisted diagnostic systems significantly outperformed traditional methods in identifying subtle fractures, showcasing the technology’s potential to enhance diagnostic workflows.

Furthermore, the integration of AI algorithms with advanced imaging techniques such as 3D CT and MRI has led to even higher diagnostic accuracies. AI’s ability to process and analyze complex imaging data allows it to identify minute fracture details that might be overlooked by human eyes. This capability underscores its critical role in modern radiology, offering a level of precision that enhances diagnostic confidence and accuracy [62].

The enhanced detection capability of AI not only aids in accurate diagnosis but also significantly contributes to better patient management and treatment outcomes. By providing detailed and precise imaging analysis, AI assists in formulating more effective treatment plans, leading to improved patient recovery rates. Moreover, AI-driven imaging analysis can detect subtle changes over time, which is crucial for monitoring the progression of conditions and adjusting treatments accordingly.

In particular, studies have demonstrated that AI models are crucial in emergency settings, where rapid and accurate diagnosis is essential to patient care. The ability to quickly and accurately diagnose fractures in emergency situations can make a significant difference in patient outcomes. For instance, in trauma cases where multiple injuries need to be assessed simultaneously, AI can prioritize and identify critical fractures that require immediate attention [63,64,65,66]. This prioritization is vital for ensuring timely interventions, which can prevent complications and improve survival rates.

The rise of Large Language Models (LLMs) such as OpenAI’s ChatGPT further enhances the integration of AI in medical imaging. These models can process and interpret vast amounts of medical literature and imaging data, providing comprehensive support for diagnostic and treatment decisions. LLMs can assist radiologists by summarizing findings, suggesting differential diagnoses, and even predicting potential complications based on the imaging data and patient history [67,68]. This integration of LLMs with imaging AI offers a holistic approach to patient care, combining the strengths of advanced imaging techniques with the analytical power of language models.

Overall, the integration of AI with advanced imaging technologies represents a significant advancement in medical diagnostics. It leverages the strengths of both fields to provide more accurate, efficient, and comprehensive care, ultimately enhancing patient outcomes and setting a new standard for radiological practice.

### 5.2. Benefits

The benefits of using AI techniques in bone fracture detection are manifold:

- **Accuracy improvements:** AI models provide higher accuracy in detecting fractures compared to traditional methods. Studies have shown that AI can significantly reduce the rate of missed fractures, particularly in complex cases where human error is more likely [37]. This increased accuracy ensures that patients receive timely and appropriate treatment, reducing the risk of complications.
- **Efficiency improvements:** Faster analysis and diagnosis, reducing the workload for radiologists. By automating the initial screening process, AI systems can quickly identify and flag potential fractures, allowing radiologists to focus on more complex cases [61,69]. This leads to improved workflow efficiency and shorter patient waiting times, ultimately enhancing the overall patient experience.
- **Enhanced diagnostic capabilities:** Improved ability to detect subtle fractures that might be missed by human eyes. AI algorithms are trained on vast datasets, enabling them to recognize patterns and anomalies that may not be apparent to human observers [62,70]. This capability is particularly valuable in detecting hairline fractures and other minor injuries, which can be crucial for early intervention and treatment.
- **Accessibility:** Enhanced access to diagnostic tools in remote and underserved areas. AI-powered diagnostic tools can be deployed in regions with limited access to specialist radiologists, providing high-quality diagnostic support where it is most needed [71,72]. This democratizes healthcare and ensures that patients in all locations can benefit from advanced diagnostic technologies.
- **Cost-effectiveness:** While not always discussed, the potential cost savings from faster, more accurate diagnoses and reduced need for follow-up imaging and treatments can be significant. AI applications can streamline workflow, reduce overhead costs, and enhance overall healthcare efficiency [73]. This is particularly important in resource-limited settings, where cost savings can make advanced diagnostic techniques more accessible.

### 5.3. Applications

The application of AI in medical imaging has already been deployed in various settings, and is expected to see even wider adoption in the near future [74]. Several studies provide concrete examples of AI applications in bone fracture detection, demonstrating the versatility and effectiveness of these technologies:

- **Proximal humerus fracture detection:** Studies using a deep learning model known as ResNet 152 have achieved an impressive accuracy of 96%, highlighting the model’s effectiveness in identifying shoulder fractures [33]. This application is particularly beneficial in emergency settings where quick and accurate diagnosis is critical. The ability to promptly identify proximal humerus fractures can significantly reduce the time to treatment, thereby improving patient outcomes and reducing the risk of complications.
- **Intertrochanteric hip fracture detection:** Another deep learning model, often referred to as VGG16, has demonstrated high sensitivity and specificity, with reported sensitivity of 93.9% and specificity of 97.4% [34]. The high performance of these models makes them suitable for integration into clinical workflows, facilitating early and accurate detection of hip fractures. This early detection is crucial for timely intervention, which can enhance recovery rates and reduce the burden on healthcare systems.
- **Chest radiograph interpretation:** Deep learning models have shown exceptional performance in detecting various thoracic diseases, including rib fractures [37]. The ability to accurately interpret chest radiographs is invaluable in the diagnosis of trauma patients. In emergency and trauma care settings, where timely and accurate diagnosis can be life-saving, AI-enhanced chest radiograph interpretation ensures that all injuries are promptly identified and treated.
- **Wrist fracture detection:** The Inception V3 model, another sophisticated AI tool, has been effectively utilized to detect wrist fractures, achieving high accuracy metrics [32]. This application is particularly useful in sports medicine and orthopedics, where wrist injuries are common. By providing quick and accurate diagnoses, AI tools can help manage and treat sports injuries more effectively, potentially reducing downtime for athletes and expediting their return to activity.
- **Automated reporting systems:** AI models integrated into clinical workflows have significantly improved diagnostic efficiency, as evidenced by various studies [26,43]. These systems can automatically generate detailed reports, reducing the administrative burden on radiologists and allowing them to focus more on patient care. Automated reporting also ensures consistency and reduces the risk of human error in documentation, leading to more reliable and standardized diagnostic outputs.

The rise of Generative AI is expected to accelerate the integration of AI technologies in clinical settings [53,75,76,77]. Generative AI can enhance diagnostic processes by creating realistic synthetic data for training models, generating detailed and tailored diagnostic reports, and even simulating various clinical scenarios to improve decision-making. These advancements can further streamline clinical workflows and enhance the overall quality of patient care.

These examples illustrate the broad range of applications for AI in bone fracture detection and the significant benefits they bring to clinical practice. From improving diagnostic accuracy and efficiency to enhancing patient outcomes, AI technologies are poised to revolutionize the field of medical imaging.

### 5.4. Challenges

Despite the promising results, several challenges remain:

- **Data quality and quantity:** Effective AI models require high-quality, annotated datasets, which are often scarce and expensive to produce [37]. Additionally, the diversity of training data are crucial to ensure AI models perform well across different populations and clinical scenarios. Efforts to create and share extensive, diverse datasets could help address this issue.
- **Generalizability:** AI models trained on specific datasets might not perform well with different populations or imaging methods. Ensuring these models are robust across various clinical settings remains a significant challenge [31,43]. Validating AI models in diverse clinical environments is necessary to ensure their reliability and effectiveness. This includes multicenter studies and data from various geographic and demographic backgrounds.
- **Integration into clinical workflows:** Integrating AI models into current clinical workflows involves overcoming technical, regulatory, and acceptance barriers. It is crucial to ensure smooth integration without disrupting clinical practices [45,46]. This includes technical integration and training healthcare providers to use AI tools effectively and trust their outputs. Successful integration requires collaboration between AI developers, healthcare providers, and regulatory authorities.
- **Ethical and legal considerations:** The use of AI in healthcare raises ethical and legal issues, including patient privacy, data security, and potential biases in AI algorithms. Addressing these concerns is essential for the broad adoption of AI technologies in medical diagnostics [28]. Regulatory frameworks must be established to ensure AI applications meet legal standards and ethical guidelines. Transparency and accountability in AI decision-making processes are also crucial for maintaining trust.
- **User acceptance:** For AI technologies to be successfully implemented, healthcare professionals must accept them. Training and education are necessary to build trust and confidence in AI-assisted diagnostic tools [78]. Resistance to change and unfamiliarity with AI technologies can impede adoption. Strategies to increase user acceptance include demonstrating the reliability and effectiveness of AI tools through clinical trials and providing ongoing education and support to healthcare providers.
- **Technical challenges:** Variations in imaging protocols and quality can impact the performance of AI models. Ensuring consistency in image acquisition and addressing technical variations are essential for reliable AI performance [33]. Developing robust AI models capable of handling a wide range of imaging conditions and integrating quality control measures into AI workflows can help mitigate these challenges.

### 5.5. Research Contributions

This review makes several significant research contributions:

- **Comprehensive synthesis:** this review provides a detailed synthesis of data from various studies, offering a thorough overview of the current state of AI in bone fracture detection, focusing on its effectiveness, benefits, applications, and challenges.
- **Identification of key trends:** it highlights key trends in AI research, such as the growing use of deep learning models like CNN, ResNet, and VGG16, which show superior performance in various fracture detection tasks.
- **Emphasis on data quality:** the review underscores the essential role of high-quality, annotated datasets in training effective AI models and the need for initiatives to create and share extensive, diverse datasets.
- **Addressing generalizability issues:** by discussing the challenges related to the generalizability of AI models, the review emphasizes the importance of validating AI models in diverse clinical settings to ensure their reliability and effectiveness.
- **Focus on integration and acceptance:** the review highlights the importance of seamlessly integrating AI into clinical workflows and the need for training and education to build trust and confidence in AI-assisted diagnostic tools among healthcare professionals.
- **Ethical and legal considerations:** by addressing ethical and legal considerations, the review calls for the establishment of regulatory frameworks to ensure that AI applications comply with legal standards and ethical guidelines, ensuring transparency and accountability in AI decision-making processes.
- **Future research directions:** the review suggests future research directions, including further validation studies, improving data quality, enhancing model robustness, facilitating seamless integration into clinical workflows, and addressing ethical and legal concerns.

### 5.6. Limitations

While this systematic review provides valuable insights into the applications of AI in bone fracture detection, several limitations should be acknowledged.

- **Database selection:** The review was conducted using specific databases recommended for their strong indexing of high-impact, high-quality articles in healthcare and information technology. However, the exclusion of broad multidisciplinary databases like WoS and Scopus may have led to the omission of some relevant studies. Future research could benefit from a more comprehensive search strategy that includes these databases to ensure broader coverage of the literature.
- **Search strategy and keywords:** The search strategy primarily focused on general AI and machine learning terms, such as “Artificial Intelligence” and “Machine Learning,” without including specific algorithms like Neural Networks, Decision Trees, Random Forests, KNN, and Bayes. This was an intentional decision to capture a broad spectrum of AI applications rather than narrow the focus to incremental performance improvements of specific models. However, this approach may have led to the exclusion of studies that focus on the detailed performance of particular machine learning techniques. Future reviews could consider incorporating these specific terms to ensure a more exhaustive collection of relevant studies.
- **Study heterogeneity:** The included studies vary significantly in terms of sample size, imaging modalities, and AI models used. This heterogeneity makes it challenging to directly compare results across studies. The lack of standardized reporting on AI model performance and the diverse clinical settings may also affect the generalizability of the findings.
- **Lack of meta-analysis:** Unlike some other systematic reviews, this study did not perform a meta-analysis due to the variability in study designs and outcome measures. This limits the ability to quantitatively synthesize the results and draw more definitive conclusions about the overall effectiveness of AI in bone fracture detection.
- **Rapidly evolving field:** The field of AI in medical imaging is rapidly evolving, with new techniques and models being developed continuously. As a result, some of the findings presented in this review may become outdated as newer studies are published. Continuous updates to the review are necessary to keep pace with the latest advancements.

## 6. Conclusions

This systematic review synthesized the current research on AI-based applications for bone fracture detection using medical images. The findings highlight the high effectiveness and potential of AI technologies in improving diagnostic accuracy and efficiency across various fracture types and imaging modalities. Studies have shown that advanced AI models, such as convolutional neural networks (CNNs) like InceptionNet, VGG16, and ResNet, often surpass human radiologists in accuracy, sensitivity, and specificity [33,34,42]. These models have been successfully applied to detect and classify proximal humerus fractures, hip fractures, chest radiographs, and dental fractures [33].

The integration of AI into clinical workflows shows promise in reducing the workload for radiologists and increasing diagnostic throughput, especially through automated reporting systems. Additionally, AI applications have effectively localized fracture sites and segmented bones in 3D imaging modalities, aiding clinicians in diagnosis and treatment planning.

However, several challenges must be addressed to fully realize the benefits of AI in healthcare. High-quality, annotated datasets are crucial for training effective AI models, but their availability is often limited, and obtaining such datasets can be costly and time-consuming [37]. Ensuring the generalizability of AI models across diverse populations and imaging modalities remains a continuous challenge, as models trained on specific datasets may not perform well in different clinical settings [31,43].

Integrating AI models into existing clinical workflows requires overcoming technical, regulatory, and acceptance barriers. Ensuring seamless integration without disrupting clinical practices is vital for the widespread adoption of AI technologies. Additionally, ethical and legal considerations, including patient privacy, data security, and potential biases in AI algorithms, must be addressed to ensure the responsible deployment of AI in healthcare [79,80].

In conclusion, while AI technologies hold significant promise for advancing medical diagnostics, particularly in bone fracture detection, concerted efforts are needed to address existing challenges. Future research should focus on improving data quality, enhancing model robustness and generalizability, facilitating seamless integration into clinical workflows, and addressing ethical and legal concerns. By tackling these issues, we can fully leverage the potential of AI to transform healthcare and improve patient outcomes.

## Figures and Tables

**Table 1:** Table contains a list of keywords used to search for articles in each database.

Database	Query Strings
Academic Search Complete	(“Bone Fracture” OR “Fracture”) AND (“Detection” OR “Diagnosis”) AND (“X-ray” AND “Diagnostic Performance”) OR (“CT scans”)
Applied Science & Technology Source	(“Bone Fracture” OR “Fracture”) AND (“Detection” OR “Diagnosis”) AND (“Machine Learning” OR “Deep Learning” OR “Imaging”)
Springer Nature Journals	(“Bone Fracture” OR “Fracture”) AND (“Detection” OR “Diagnosis”) AND (“Machine Learning” OR “Deep Learning” OR “Imaging”)
ScienceDirect	(“Bone Fracture” OR “Fracture”) AND (“Detection” OR “Diagnosis”) AND (“Machine Learning”)
Journals@OVID	(“Fracture Diagnosis” AND “Imaging” AND Bone Fracture)
Directory of Open Access Journals	(“Bone Fracture” OR “Fracture”) AND (“Detection” OR “Diagnosis”)
Radiological Society of North America	(“Bone Fracture” OR “Fracture”) AND (“Detection” OR “Diagnosis”) AND (“Machine Learning”)
MEDLINE	(“Bone Fracture” OR “Fracture”) AND (“Detection” OR “Diagnosis”) AND (“X-ray” AND “Diagnostic Performance”) AND (“CT scans”)
JSTOR Journals	(“Fracture Diagnosis” AND “Imaging” AND Bone Fracture)

**Table 2:** This table contains the data we extracted from each paper selected in this review study. Some data were not found in every paper.

Data Item	Description
**Title**	Title of the paper
**Author(s)**	Author name(s)
**Type**	e.g., conference/workshop/journal
**Date**	Publishing year
**Country**	Country of authors
**Study Design**	Type of study (e.g., retrospective, prospective)
**Sample Size**	Number of subjects or images analyzed
**AI Technique Used**	Specific AI methods (e.g., deep learning, machine learning algorithms)
**Performance Metrics**	Accuracy, sensitivity, specificity, precision, recall, F1 score, ROC-AUC
**Comparison with Other Methods**	Performance compared to traditional methods or other AI techniques
**Validation Methods**	Cross-validation, external validation, or use of independent test sets
**Statistical Significance**	P-values or confidence intervals to determine significance
**Efficiency Improvements**	Speed of detection, time savings for radiologists
**Accuracy Improvements**	Increased diagnostic accuracy, reduction in human error
**Cost-Effectiveness**	Cost savings in the diagnostic process
**Accessibility**	Improved access to diagnostics in remote or underserved areas
**Enhanced Diagnostic Capabilities**	Detection of fractures that are difficult to identify with traditional methods
**Software Applications**	Names and descriptions of software developed
**Commercial Products**	AI-based products available on the market
**Integration with Medical Systems**	How applications integrate with existing medical imaging systems (e.g., PACS, RIS)
**Use Cases**	Examples of applications in clinical practice
**Technical Challenges**	Issues related to algorithm performance, data quality, and computational requirements
**Regulatory and Ethical Issues**	Regulatory hurdles, ethical concerns regarding AI use in healthcare
**User Acceptance**	Acceptance and trust by medical professionals
**Integration Challenges**	Difficulty in integrating AI applications with existing hospital systems
**Data Privacy and Security**	Concerns about patient data privacy and cybersecurity
**Cost and Resource Requirements**	Costs of implementation, need for specialized resources or training
**Future Areas**	Areas of future investigation
**Comments**	Remarks about the quality of the paper

**Table 3:** Comparison of various studies on AI-based fracture detection across different skeletal joints. The table summarizes the dataset characteristics, type of images used, models applied, skeletal joints targeted, study descriptions, performance metrics, and key remarks for each study.

No.	Reference	Year	Dataset	Modality	Model	Parts	Description	Performance
**1**	Olczak et al. [59]	2017	The dataset consists of 256,000 radiographs of the wrist, hand, and ankle.	Radiographic images	AlexNet, NIN, VGGs	Various Parts	This research demonstrates that deep learning can exceed human performance.	Accuracy = 0.83
**2**	Kim et al. [32]	2018	There are 695 wrist radiographs with fractures and 694 without fractures.	Radiographic images	Inception V3	Wrist	The author validated that using transfer learning from CNNs for fracture detection on radiographs can achieve top-tier performance.	AUC = 95.4, Sensitivity = 90, Specificity = 88
**3**	Chung et al. [33]	2018	The dataset includes 1891 plain shoulder AP radiographs (1376 with proximal humerus fractures and 515 normal shoulders) from 1891 patients, comprising 591 men and 1300 women.	Radiographic images	Resnet 152	Humeral	The authors introduced a model for identifying and categorizing fractures from AP shoulder radiographic images.	Accuracy = 96, Sensitivity = 0.99, Specificity = 0.97, AUC = 0.996
**4**	Urakawa et al. [34]	2018	The dataset includes 3346 hip images, with 1773 fractured and 1573 non-fractured, collected from the Department of Orthopedic Surgery, Tsuruo.	Radiographic images	VGG16	Hips	This study presents a performance comparison between CNNs and orthopedic surgeons.	Accuracy = 95.5, Sensitivity = 93.9, Specificity = 97.40, AUC = 0.984
**5**	Yu et al. [35]	2019	There are 307 patients with APFFs and 310 normal patients.	Radiographic images	InceptionV3	Hips	The proposed algorithm excelled in detecting APFF but struggled with precise fracture localization.	Accuracy = 96.9, AUC = 0.994, Sensitivity = 97.1, Specificity = 96.7
**6**	Choi et al. [36]	2019	The dataset comprises 1266 pairs of AP and lateral elbow radiographs examined between January 2013 and December 2017 at a single institution.	Radiographic images	Resnet 50	Elbow	The authors focused on developing a dual-input CNN-based model for the automated detection of supracondylar fractures.	AUC = 0.985, Sensitivity = 93.9, Specificity = 92.2
**7**	Majkowska et al. [37]	2020	The study utilized two datasets: DS1 with 759,611 images from a multicity hospital network, and ChestX-ray14, a publicly available dataset with 112,120 images. Natural language processing and expert review labeled 657,954 training images. The test sets included 1818 images from DS1 and 1962 from ChestX-ray14.	Radiographic images	Xception	Chest	The authors created a model to identify opacity, pneumothorax, mass or nodule, and fractures.	AUC = 0.86, Sensitivity = 59.9, Specificity = 99.4
**8**	Johari et al. [38]	2016	The dataset contains 240 radiographs of teeth: 120 with no VRFs and 120 with vertical fractures. Each category is split equally between endodontically treated and untreated teeth.	Radiographic images	Probabilistic neural network (PNN) CBCT-G1/2/3, PA-G1/2/3	Vertical Roots	This study supports the preliminary detection of vertical root fractures.	Accuracy = 96.6, Sensitivity = 93.3, Specificity = 100, CMPIs THRESHOLD = 0.79
**9**	Heimer et al. [39]	2018	The dataset includes 84 skull fracture cases, with 5 excluded due to severe destruction and 4 removed because of surgical material. For each of the 75 included cases, a corresponding case without documented skull fractures was retrieved.	Images are extracted from Postmortem computed tomography (PMCT)	Deep neural networks	Skull	The study aims to classify and detect skull fractures using curved maximum intensity projections (CMIP) and deep neural networks.	Specificity = 87.5, Sensitivity = 91.4, CMPIs THRESHOLD = 0.75
**10**	Kitamura et al. [40]	2019	There are 298 normal and 298 fractured ankle studies identified by parsing radiology reports.	Radiographic images	Seven models: Inception V3, ResNet, Xception	Ankle	The study evaluated the efficiency of CNNs on small datasets.	Best performance by Ensemble_A, Accuracy = 83, Sensitivity = 80, Specificity = 81
**11**	Gan et al. [41]	2019	The training dataset includes 2040 images (1341 with DRFs and 699 without DRFs) and a test dataset of 300 images (150 with DRFs and 150 without DRFs).	Radiographic images	Inception V4	Wrist	The authors implemented an algorithm to detect distal radius fractures.	Accuracy = 93, AUC = 0.961, Sensitivity = 90, Specificity = 96
**12**	Lindsey et al. [42]	2018	The dataset comprises 135,845 radiographs of various body parts. The remaining 100,855 radiographs cover 11 body parts. The shoulder has the most radiographs (26,042), while the spine has the least (885).	Radiographic images	Unet	Wrist	This study involves using deep learning to help doctors distinguish between fractured and normal wrists.	AUC = 97.5%, Sensitivity = 93.9%, Specificity = 94.5%
**13**	Adams et al. [43]	2019	Various dataset sizes (200, 320, and 640 images) are split into training (80%) and validation (20%), with an additional 160 images used as the final test set.	Radiographic images	AlexNet and GoogLeNet	Femur	The author aimed to assess the accuracy of DCNN for femur fracture detection.	Accuracy AlexNet = 89.4%, GoogLeNet = 94.4%
**14**	Rahmaniar et al. [44]	2018	The dataset includes 815 coronal images, 777 transverse images, and 618 sagittal images.	A computerized tomography (CT) images	Computerized system	Calcaneal Fractures	In this study, the author focused on detecting femoral neck fractures using genetic and deep learning methods.	Accuracy = 0.86, precision rate = 0.86, recall = 0.89
**15**	Tomita et al. [45]	2018	The dataset consists of 1432 CT scans, comprising 10,546 two-dimensional images in sagittal view, with a test set of 129 CT scans.	A computerized tomography (CT) images	Deep convolutional neural network (CNN)	vertebra	This study aims at the early detection of osteoporotic vertebral fractures.	Accuracy = 89.2%, F1 score = 90.8%, sensitivity = 85.2%, specificity = 95.8%
**16**	Muehlematter et al. [46]	2019	Standard CT scans of 58 patients with insufficiency fractures of the spine were performed between 2006 and 2013.	A computerized tomography (CT) images	Machine-learning algorithms	vertebra	The author aimed to evaluate the performance of bone texture analysis with a machine learning algorithm.	AUC = 0.64
**17**	Wang et al. [47]	2022	The dataset includes 222 training images, 56 validation images, and 408 testing images of CT scans.	A computerized tomography (CT) images	U-Net and ResNet	Mandibule	The author developed a novel method for classifying and detecting mandibular fractures.	Accuracy = 90%, AUC = 0.956
**18**	Pranata et al. [48]	2019	Two datasets were used: the first contains 255 fractured and 732 normal images (totaling 987), and the second includes 428 fractured and 516 normal images (totaling 944).	A computerized tomography (CT) images	ResNet and VGG	Femoral Neck	The author aimed at detecting femoral neck fractures using genetic and deep learning techniques.	Accuracy = 0.793, Specificity = 0.729, Sensitivity = 0.829
**19**	Cheng et al. [60]	2019	The dataset comprises 25,505 hip radiographs.	Radiographic images	DenseNet-121	limb	The goal of this study was to localize and classify hip fractures using deep learning.	accuracy = 91%, sensitivity = 98%, false-negative rate = 2%, AUC = 0.98

**Table 4:** Publication year distribution of selected studies.

Year Range	Number of Publications
2016–2017	2
2018–2019	13
2020–2021	1
2022–2023	1

**Table 5:** Geographic distribution of selected studies.

Country	Number of Publications
United States	6
China	4
Japan	3
United Kingdom	2
Iran	2
Switzerland	2
India	1
Australia	1
South Korea	1

**Table 6:** Publication types of selected studies.

Type	Number of Publications
Journal	18
Conference	2

**Table 7:** Top journals and conferences of selected studies.

Journal/Conference Name	Number of Publications
*Diagnostics*	2
*Clinical Radiology*	2
*Acta Orthopaedica*	2
*Skeletal Radiology*	1
*Dentomaxillofacial Radiology*	1
*Computers in Biology and Medicine*	1
*European Radiology*	1
*Clinical Oral Investigations*	1
*Journal of Medical Imaging and Radiation Oncology*	1
*IEEE Transactions on Medical Imaging*	1
*Proceedings of the National Academy of Sciences*	1
*Journal of Medical Imaging*	1
*Radiology*	1
*Artificial Intelligence in Medicine*	1

## Data Availability

No new data were created or analyzed in this study.
